# Identification of the first endolysin Cell Binding Domain (CBD) targeting *Paenibacillus larvae*

**DOI:** 10.1038/s41598-019-39097-2

**Published:** 2019-02-22

**Authors:** Sílvio B. Santos, Ana Oliveira, Luís D. R. Melo, Joana Azeredo

**Affiliations:** CEB - Centre of Biological Engineering, LIBRO - Laboratório de Investigação em Biofilmes Rosário Oliveira, University of Minho, 4710-057 Braga, Portugal

## Abstract

Bacteriophage endolysins present enormous biotechnological potentials and have been successfully used to control and detect bacterial pathogens. Endolysins targeting Gram-positive bacteria are modular, displaying a cell binding (CBD) and an enzymatically active domain. The CBD of phage endolysins are recognized by their high specificity and host affinity, characteristics that make them promising diagnostic tools. No CBD able to bind *Paenibacillus larvae* has been identified so far. *P*. *larvae* is a Gram-positive spore forming bacteria that causes the American Foulbrood. This highly contagious infection leads to honeybee larvae sepsis and death, resulting in an adverse impact on pollination and on the beekeeping industry. In this work, the first CBD targeting *P*. *larvae* was identified and its core binding sequence was investigated. Moreover, it was shown that the domain is highly specific, targeting exclusively *P*. *larvae* cells from all ERIC genotypes. The identification of such a domain represents a step forward for the development of effective methods to detect and control this pathogen.

## Introduction

Endolysins (lysins) are phage-encoded enzymes expressed at the end of the phage life cycle that target and cleave bonds of the host cell wall peptidoglycan, degrading the murein layer and allowing the release of new virions^1–3^. Lysins from a Gram-positive background usually show a modular structure, composed of two functional domains: the enzymatically active or catalytic domain (EAD) and the cell wall binding domain (CBD). The latter confers specificity to these enzymes targeting specific bonds of the cell wall surface (*e*.*g*. peptidoglycan, secondary cell wall polysaccharides and proteins^2–4^). This specificity is usually very narrow, going from a specific genus until the strain level with a binding affinity that is equal to or higher than that of antibodies to their antigens^2^. As a consequence of this high specificity, fluorescently labelled CBDs have been proposed as alternative tools for the detection of microorganisms^4,5^. Moreover, CBDs do not suffer from the usually drawbacks of antibodies: difficult, expensive, and time-consuming production; sensitivity to temperature and pH; and propensity  to aggregation^6–8^.

Their intrinsic characteristics together with the successful results obtained enabled CBDs to emerge as promising diagnostic tools^4,5^. Kretzer and colleagues were able to detect 1 to 100 CFU of *Listeria* per gram of food within 20–40 min, with an enrichment step of 6 h^9^. Efficient detection of *Bacillus cereus* and *Clostridium perfringens* (and others) was also achieved with phage CBDs.

Honeybees are compromised by many pathogens such as bacteria, fungi, viruses and parasites. One of the most devastating bacterial diseases affecting honeybee larvae of *Apis mellifera* and other *Apis* spp is the American Foulbrood (AFB). AFB is a highly contagious and lethal disease caused by *Paenibacillus larvae*, a worldwide-distributed spore-forming Gram-positive bacterium that affects honeybee larvae^10^.

Currently, the only effective treatment and control measure for the AFB is the incineration of the infected hives and contaminated equipment, which causes important economic losses in the sector^11,12^. Antibiotics for the control of bee diseases were banned in many countries (Regulation (EEC) 2377/90 and further amendments applies in the European context) as their use induced bacterial resistances and promoted the presence of relatively long half-life antibiotic residues in raw honey affecting the honey quality and safety for human consumption^10,11,13,14^. The economic impact in the beekeeping industry and the critical role of honeybees in crop pollination^10,15^ encourages the development of new strategies for the detection and control of AFB.

Molecular methods, as those based on PCR, have overcome many of the cultivation-based limitations to detect bacteria due to their specificity, high sensitivity and enrichment culturing avoidance. However, the efficiency of DNA recovery from samples, the influence of inhibitors in the PCR reaction derived from the sample matrix and the false-positive results obtained from DNA of dead bacteria can compromise the success of such methods^16^. The identification of new proteins as bio-recognition elements able to specifically bind to this bacterium will play a critical role in the design of new detection and control methods for *P*. *larvae*.

So far, there are no known CBDs able to bind *P*. *larvae.* Their identification would enable not only to develop new detection methods but also to design new drugs specific for this problematic bacterium. The genome annotation of the previously isolated *P*. *larvae* phage phiIBB_Pl23 enabled the identification of its lysin (PlyPl23) with a conserved catalytic domain at its N-terminus but with no detectable domain at the C-terminus. The Gram-positive nature of the lysin led us to hypothesize the existence of a novel CBD^17^.

In this work we aimed at identifying the first lysin CBD able to specifically bind to *P*. *larvae*. To accomplish that we performed a functional analysis of the binding ability of the protein’s C-terminus, assessed its specificity and identified the CBD sequence (considered the smallest peptide sequence able to preserve its binding activity).

## Results

### Bioinformatic analysis

The previous bioinformatics analysis (in 2015) of the *P*. *larvae* phage phiIBB_Pl23 genome^17^ predicted the existence of a lysin with a N-terminal Amidase_2 domain but was unable to identify a binding domain at the enzyme C-terminus. Nevertheless, considering the Gram-positive nature of the PlyPl23 lysin, we hypothesized the existence of a novel CBD.

**Figure 1:**
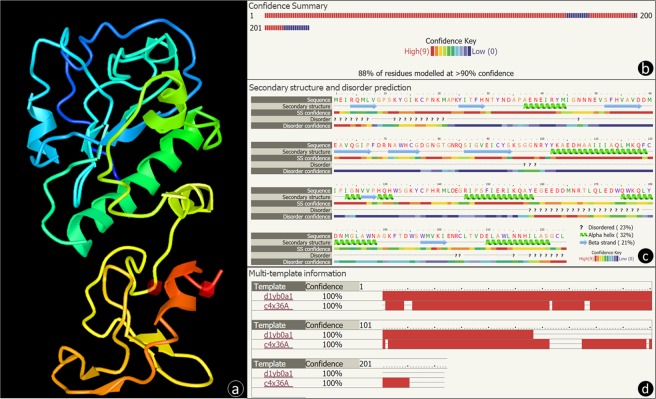
Predicted protein structure of PlyPl23 lysin. The protein structure of PlyPl23 was predicted using Phyre2^33^. (**a**) A 3D model, ribbon diagram, coloured by rainbow N to C terminus of PlyPl23, showing the two separated functional domains (EAD at the top and CBD at the bottom) connected by a linker (beginning of the yellow colour). The red cubes correspond to residues E161 and C223 (ahead identified as the beginning and the end of the CBD). (**b**) Colour-coded confidence summary of the predicted model by residue showing that 88% of the residues were modelled with more than 90% confidence. (**c**) Predicted secondary structure (alpha helixes and beta strands) and disordered regions, colour coded by confidence level. (**d**) Multi-template information for the modelled protein structure. Two templates were selected to model the protein based on heuristics to maximise confidence, percentage identity and alignment coverage. The table indicates where the sequence was covered by each template, colour-coded by the confidence of the match to that template overall. 27 residues were modelled by *ab initio* which is highly unreliable.

A 3D model of the protein structure was obtained using Phyre2 (Fig. 1a), with 88% of residues modeled and a level of confidence higher than 90% (templates with the fold library id d1yb0a1 and c4x36A were used) (Fig. 1b,d). The predicted protein 3D structure (Fig. 1a) clearly shows the existence of two different domains connected by a linker (beginning of the yellow color), and the first domain (top) clearly encloses the sequence corresponding to the predicted N-terminal catalytic domain. The second domain (bottom) starts with a disordered region (a region that lacks a fixed or ordered three-dimensional structure), followed by an alpha helix, a beta strand and another alpha helix, and it ends with a small disordered region (Fig. 1c).

### Functional analysis and specificity of the lysin C-terminus

**Figure 2:**
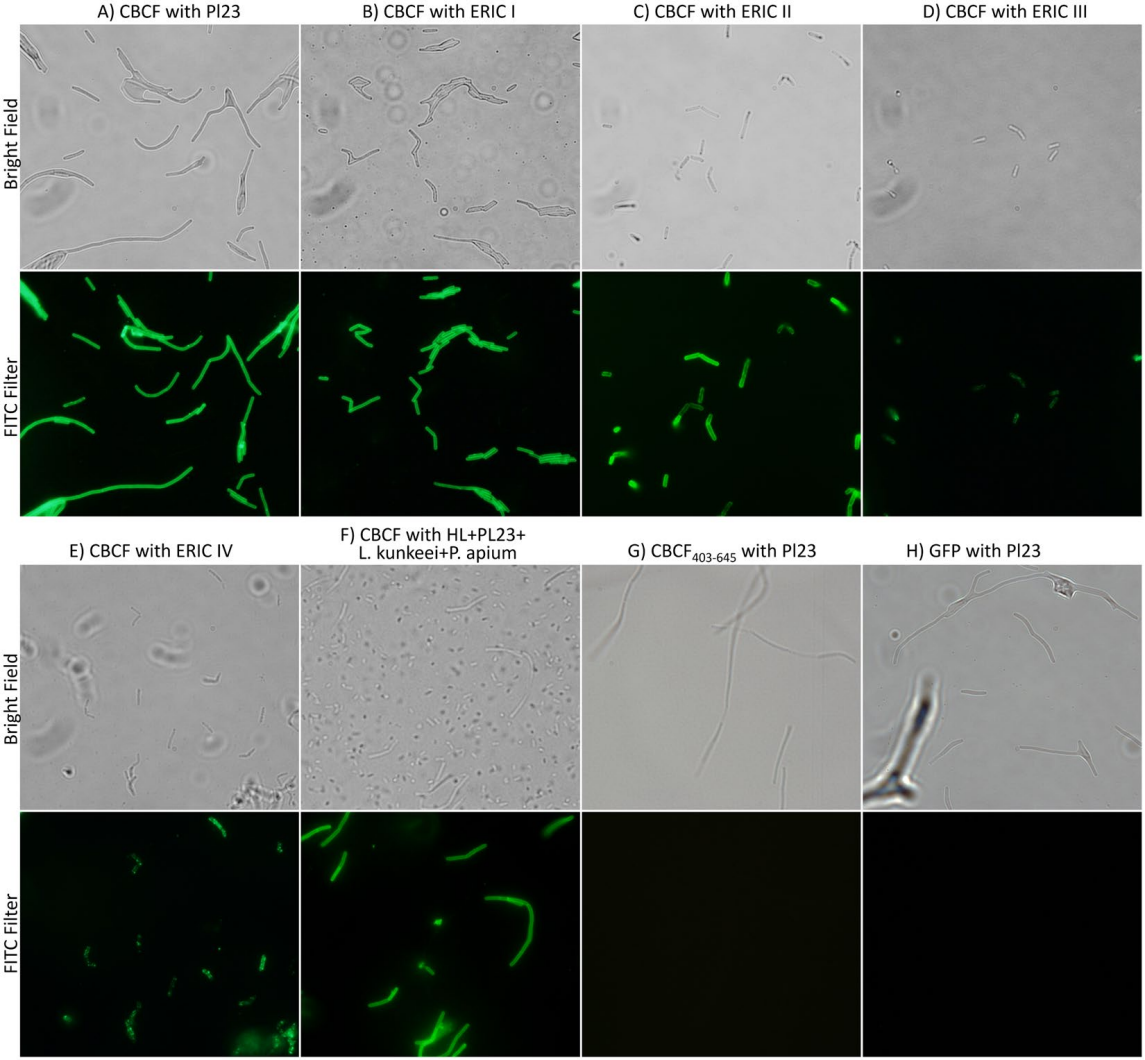
Fluorescence microscopy of the different *P*. *larvae* cells decorated with the GFP-CBCF. The different *P*. *larvae* ERIC genotypes were incubated with the fusion protein GFP-CBCF, GFP-CBCF_403–645_ (truncated CBCF fragment from nucleotide 403 to 645) or GFP only, and observed at the fluorescence microscope to assess the specificity of their binding ability to these bacterial cells: (**A**) GFP-CBCF with *P*. *larvae* Pl02-23 (the phage host) that belongs to ERIC *I*; (**B**) GFP-CBCF with another *P*. *larvae* ERIC *I*; (**C**) GFP-CBCF with *P*. *larvae* ERIC *II*; (**D**) GFP-CBCF with *P*. *larvae* ERIC *III*; (**E**) GFP-CBCF with *P*. *larvae* ERIC *IV*; (**F**) GFP-CBCF with homogenised larvae (HL) artificially contaminated with *P*. *larvae* Pl02-23, *Lactobacillus kunkeei* (LMG 18925) and *Parasaccharibacter apium* alpha 2.2; (**G**) GFP-CBCF_403–645_ with *P*. *larvae* Pl02-23; (**H**) GFP with *P*. *larvae* Pl02-23. Observations were made in bright field and under FITC to detect the presence of non-decorated cells. White bar scale represents 10 µm.

To prove the existence of a CBD we cloned the C-terminus fragment of PlyPl23 from base 403 to 675 (corresponding to residues K135 to L224), called herein as cell binding-containing fragment (CBCF). The fragment ends were selected to assure that the fragment would accommodate the hypothesized CBD. The peptide was fused to a green fluorescent protein (GFP) originating the recombinant protein GFP-CBCF. After incubating the GFP-CBCF with *P*. *larvae* cells, fluorescent microscope observations revealed green-decorated cells (Fig. 2). On the other hand, functional analysis of the truncated PlyPl23 catalytic domain revealed no lytic activity on *P*. *larvae* (data not shown). The binding specificity of PlyPl23 CBD was assessed through the ability of GFP-CBCF to decorate different *P*. *larvae* strains from different ERIC genotypes (*I* to *IV*) and other related bacterial species. Fluorescence microscopy observations revealed that the GFP-CBCF was able to decorate all the tested *P*. *larvae* cells (Fig. 2A,E) but not those from non-*P*. *larvae* strains (Fig. 2F). Furthermore, GFP alone was not able to decorate any of the tested strains, including the phage host *P*. *larvae* Pl02-23 (Fig. 2H).

Homogenized larvae (HL) artificially contaminated with *P. larvae*, *Lactobacillus kunkeei* and *Parasaccharibacter apium* was used to simulate field conditions and to assess possible interferences of this matrix in CBD binding. Fluorescence microscope observations showed that the incubation of the GFP-CBCF with the HL still enables the specific decoration of all *P*. *larvae* cells, easily recognized by their typical shape in bright field (Fig. 2F).

### Identification of the CBD

Aiming at identifying the PlyPl23 CBD, considered the smallest peptide that still retains the binding ability, several truncations of the CBCF were carried out (using the primers presented in Table 1), firstly on the CBCF N-terminus (maintaining the end of the CBCF) and then on the C-terminus (maintaining the beginning of the CBCF). The expressed CBCF truncated peptides were tested for binding activity. Fluorescence microscopy observations revealed that when we truncated the CBCF fragment at residue 161 or at residue 223 its binding ability was maintained (Table 1). However, when one residue at the N or C-terminus was further removed, the fragment was no longer able to bind to the ERIC *IV* strain. Consequently, the fragment containing residues E161 to C223 was constructed and its binding ability to the *P*. *larvae* strains was assessed. The results showed that this fragment maintained the same binding ability as the CBCF (K135 to L224). The complete GFP-CBCF was used as a positive control (Fig. 2A,E) while GFP alone and the truncated CBCF fragment CBCF_403–645_ (from nucleotide 403 to 645, residues K135 to N215) was used as a negative control (Fig. 2H,G respectively).

**Table 1:** Primers used to perform the several truncations of the CBCF (to identify the CBD) and binding ability of the corresponding truncations.

Name	RE	Primer Sequence	Tm	AA	CBCF
Pl23Fw403	SacI	AGAAGCGAGCTCAAATACTGCCCGCACAGAATGTTAG	56.0	135	
Pl23Fw433	SacI	GCCGCCGAGCTCGGAAGAATACCAAGCTTTATAGAGC	54.4	145	
Pl23Fw463	SacI	GCCGCCGAGCTCAAACAAGCATACGAAGGAGAGGAAG	56.0	155	
Pl23Fw466	SacI	GCCGCCGAGCTCCAAGCATACGAAGGAGAGGAAG	54.8	156	
Pl23Fw469	SacI	GCCGCCGAGCTCGCATACGAAGGAGAGGAAGAC	54.4	157	
Pl23Fw472	SacI	GCCGCCGAGCTCTACGAAGGAGAGGAAGACGACA	54.8	158	
Pl23Fw475	SacI	GCCGCCGAGCTCGAAGGAGAGGAAGACGACATGA	54.8	159	
Pl23Fw478	SacI	GCCGCCGAGCTCGGAGAGGAAGACGACATGAATAG	55.3	160	
Pl23Fw481	SacI	GCCGCCGAGCTCGAGGAAGACGACATGAATAGAACC	55.7	161	
Pl23Fw484	SacI	GCCGCCGAGCTCGAAGACGACATGAATAGAACCTTACA	54.8	162	
Pl23Fw487	SacI	GCCGCCGAGCTCGACGACATGAATAGAACCTTACAACT	54.8	163	
Pl23Fw490	SacI	GCCGCCGAGCTCGACATGAATAGAACCTTACAACTGGA	54.8	164	
Pl23Fw493	SacI	GCCGCCGAGCTCATGAATAGAACCTTACAACTGGAAGATT	54.1	165	
Pl23Fw496	SacI	GCCGCCGAGCTCAATAGAACCTTACAACTGGAAGATTGG	55.2	166	
Pl23Fw499	SacI	GCCGCCGAGCTCAGAACCTTACAACTGGAAGATTGG	54.0	167	
Pl23Fw502	SacI	GCCGCCGAGCTCACCTTACAACTGGAAGATTGGCAAT	54.0	168	
Pl23Fw505	SacI	GCCGCCGAGCTCTTACAACTGGAAGATTGGCAATGGAA	54.8	169	
Pl23Fw520	SacI	GCCGCCGAGCTCTGGCAATGGAAACAGCTCTATGA	53.5	174	
Pl23Rv540	XhoI	CCGCCGCTCGAGATAGAGCTGTTTCCATTGCCAATC	54.0	180	
Pl23Rv600	XhoI	CCGCCGCTCGAGCTTGACCATCCAGCTCCAATC	54.4	200	
Pl23Rv615	XhoI	CCGCCGCTCGAGTCAACAACGATTTTCAATCTTGACCATCC	54.8	205	
Pl23Rv630	XhoI	CCGCCGCTCGAGTCACTCATCAACGGTAAGACAACGATTTT	54.8	210	
Pl23Rv645	XhoI	CCGCCGCTCGAGTCAATTCAACCATGCCAGCTCATCAA	53.5	215	
Pl23Rv648	XhoI	CCGCCGCTCGAGTCAGTTATTCAACCATGCCAGCTCATC	55.7	216	
Pl23Rv651	XhoI	CCGCCGCTCGAGTCAGTGGTTATTCAACCATGCCAGC	54.8	217	
Pl23Rv654	XhoI	CCGCCGCTCGAGTCAAATGTGGTTATTCAACCATGCCAG	54.0	218	
Pl23Rv657	XhoI	CCGCCGCTCGAGCAAAATGTGGTTATTCAACCATGCC	54.4	219	
Pl23Rv660	XhoI	CCGCCGCTCGAGTCACGCCAAAATGTGGTTATTCAACCAT	54.4	220	
Pl23Rv663	XhoI	CCGCCGCTCGAGTCAACTCGCCAAAATGTGGTTATTCAAC	54.4	221	
Pl23Rv666	XhoI	CCGCCGCTCGAGTCACCCACTCGCCAAAATGTGGTTA	54.8	222	
Pl23Rv669	XhoI	CCGCCGCTCGAGTCAGCACCCACTCGCCAAAATGT	53.8	223	
Pl23Rv672	XhoI	CCGCCGCTCGAGTCACAGGCACCCACTCGCCAA	54.9	224	
Pl23Rv675	XhoI	TACGATCTCGAGTCACAGGCACCCACTCGC	54.9	225	

Primers containing Fw or Rv on their name correspond to primers designed in the forward or the reverse strand respectively. RE – restriction enzyme sequence included in primer design. Tm – melting temperature of the primer calculated through OligoCalc^35^. AA – boundary amino acid targeted by the primer. CBCF (cell binding containing fragment) – schematic representation of the amplified fragments (with the corresponding Fw primer until the end of the CBCF (Pl23Rv675) or the beginning of the CBCF (Pl23Fw403) and the corresponding Rv primer) related to the CBCF. (+) means that the amplified fragment decorated the *P*. *larvae* cells while (−) means that the fragment did not bind to *P*. *larvae*.

### Homologs and multiple sequence alignment

**Figure 3:**
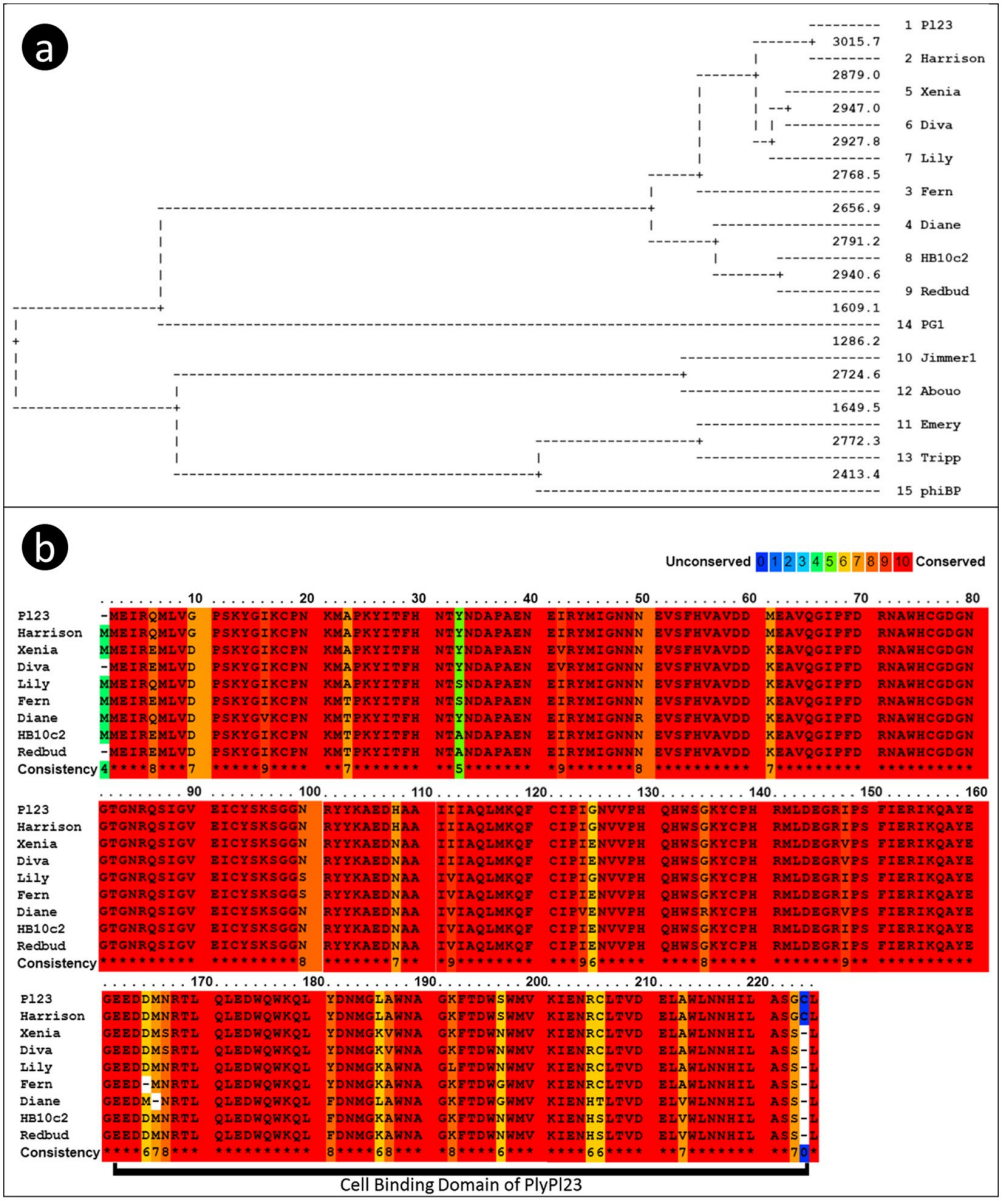
Multiple sequence alignment of the *Paenibacillus* phage lysins using Praline^18^. (**a**) Tree diagram of the alignment with all the *Paenibacillus* phage lysins; (**b**) Colour-coded scheme for amino acid conservation of the well conserved group composed of lysins from 9 phages (which include PlyPl23). The scoring scheme works from 0 for the least conserved alignment position, up to 10 for the most conserved alignment position.

**Figure 4:**
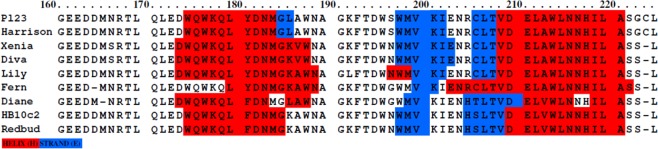
Secondary structure colour-coded scheme of the CBD alignment of the well conserved group composed of lysins from 9 phages using Praline^18^ showing the differences in peptide sequence and its impact in the predicted structure of the lysins CBD.

A BLASTp analysis revealed 17 *P*. *larvae* phage lysins homologs to PlyPl23 but none were found within lysins affecting other *Bacillus* species. A search at the NCBI for other *Paenibacillus* phages returned seven additional *P*. *larvae* phage genomes (which do not present homologs to PlyPl23) as well as a *P*. *polymyxa* phage genome. The annotated lysins were retrieved accounting for a total of 15 different lysins from the 25 different proteomes of phages infecting *Paenibacillus* genus. Phages with identical lysins are: Harrison = Paisley, Fern = Willow, Diva = Sitara = Shelly, Diane = Hayley = Vegas = Vadim, HB10c2 = Rani, Jimmer1 = Jimmer2 and Abouo = Davies. A multiple sequence alignment of all the fetched *Paenibacillus* phage lysins using Praline^18^ is presented in Fig. 3. The tree representation of the alignment shows two distinct groups of lysins (Fig. 3a), including the PlyPl23 lysin in a consistent group of 9 lysins. A closer look on the alignment of the PlyPl23 lysin group (Fig. 3b) demonstrates a very well conserved sequence among the 9 lysins, not only at the N-terminal EAD but also at the C-terminal CBD. Looking into the CBD stretch (Fig. 4) it is possible to observe that some of these lysins present an equal sequence: PlyPl23 = Harrison; Xenia = Diva; and HB10c2 = Redbud. Although only point differences are observed in the CBD sequences, they are able to produce modifications in their structure as can be seen in the prediction obtained by Praline (Fig. 4).

## Discussion

In a previous work we have identified for the first time a lysin able to kill *P*. *larvae*^17^. The *in silico* analysis of its sequence did not reveal at that time any known binding domain at the C-terminus, after the amidase catalytic domain^17^. An up-to-date bioinformatic analysis of the annotated lysins from the currently sequenced 25 *Paenibacillus* phage genomes deposited at the NCBI (including PlyPl23) was still unable to identify a functional domain at their C-terminus. The prediction of two structural domains composing the PlyPl23 lysin (Fig. 1), with the first domain enclosing the sequence corresponding to the predicted N-terminal catalytic domain, reinforces the hypothesis of the existence of a second domain responsible for cell binding. The low confidence level for PlyPl23 structure prediction for residues 163–174 and 211–224 (Fig. 1b) is related to the lack of homology to known templates (Fig. 1d). These regions were also predicted to be disordered (Fig. 1c), meaning that they lack a fixed 3D structure. These disordered regions can present functional sites known as linear motifs, which may contain protein ligands (*e*.*g*. SH3 ligands). The binding of these ligands to their target can induce a disorder to order transition^19,20^. Despite the variability of CBDs that enable lysins’ specificity against their target bacterium, they usually display conserved modules (*e*.*g*. LysM, SH3b and Cpl-7), which facilitates the *in silico* identification. However, some lysins’ modules (*e*.*g*. the present case) can have different binding patterns from those already annotated that have to be identified experimentally^2^. The observation of green-decorated cells by fluorescence microscopy after incubation with the fusion protein GFP-CBCF, and the inability of GFP alone to decorate the same cells, indicates that the CBCF peptide stretch contains a CBD (Fig. 2). This corresponds to the first identification of a lysin CBD able to bind *Paenibacillus larvae* cells. The evaluation of the binding specificity of GFP-CBCF revealed that PlyPl23 CBD is able to bind to all tested *P*. *larvae* strains. This specificity was already expected since it corroborates the results obtained for the lytic spectrum of the PlyPl23 obtained before^17^. The specificity of the protein is an important feature when envisaging its potential use in the diagnosis of AFB, avoiding false positive results. Indeed, no binding occurred to other related and unrelated bacteria tested. Importantly, the bacterial species that were not susceptible to GFP-CBCF binding are those present in the microbiota of honeybee larvae, namely *Lactobacillus kunkeei* (LMG 18925) and *Parasaccharibacter apium* alpha 2.2^21^. Envisaging a possible application of this CBD in the detection of *P*. *larvae* in field samples it is important to guarantee that the sample matrix does not interfere in the measurements, which in some cases may hinder the binding of the ligand to its receptor impairing the detection of the target bacteria^22^. Results revealed no interference of homogenized larvae (HL) in the GFP-CBCF binding (Fig. 2F) guaranteeing its feasibility for field applications.

Interestingly, the binding patterns to ERIC *III* and *IV* (Fig. 2D,E) are different from those observed to the other two ERIC types (Fig. 2A,C). In fact, we observed that the fluorescence intensity is lower in ERIC *III* and *IV* and that its distribution is not homogeneous along the bacterial cell surface, with some intense dots randomly distributed along the cells, less pronounced in ERIC *III* (Fig. 2D). This indicates that the CBD receptors in the cell wall of ERIC *III* and *IV* strains are probably slight different from those of other strains and/or that they are present in lower number or less available to CBD interaction. Therefore, it is expectable that the cell wall structure (in composition and/or arrangement) of these genotype strains is different from the other *P*. *larvae* strains. In fact, LeBlanc *et al*. in 2015^23^ reported a lower activity of the lysin PlyPalA (from Xenia’s phage) against ERIC *III* and ERIC *IV* strains. Considering the high homology with PlyPl23 (Figs 3 and 4), their result can be explained by the lower affinity of the lysin’s CBD to the cell walls of ERIC types *III* and *IV*. Also, functional analysis of the truncated PlyPl23 catalytic domain revealed no lytic activity in *P*. *larvae*, indicating that the CBD is essential for lysis probably by providing the necessary proximity between the enzyme and its substrate, as suggested and observed before^2,4,24^. A similar result was obtained with the truncated phiBP catalytic domain^25^.

The several truncations of the CBCF fused to the GFP enabled the identification of the smallest peptide sequence that retains the binding ability, which corresponds to the functional *P*. *larvae* lysin’s CBD. The fragments 161–224 and 135–223 still retained the same binding affinity as the CBCF but not the fragments 162–224 and the 161–222, which were no longer able to bind to ERIC *IV* strains. Consequently, it was possible to infer that the CBD fragment starts at residue E161 and ends at residue C223, being composed by 63 amino acids. Construction and evaluation of the fragment E161-C223 confirmed this hypothesis. The lack of binding of GFP alone and of the fragment CBCF_403–645_ to *P*. *larvae* Pl02-23 show that the cell decoration is not a result of unspecific binding (Fig. 2H,G).

This newly identified CBD presents high potential to be used as a detection tool for the diagnosis of AFB due to its specificity and broad range towards *P*. *larvae* species. Moreover, its small size enables an easy production (*e*.*g*. heterologous expression in *E*. *coli* systems) and ability to be fused to other proteins for diverse applications targeting *P*. *larvae*. The conserved catalytic domain of PlyPL23 among different bacterial genus (*e*.*g*. *Bacillus*) together with the PlyPl23 specificity against *P*. *larvae* show that this CBD is the main responsible for the lysin specificity. This suggests another potential application of the CBD: its fusion with unspecific antimicrobial proteins, turning them specific for *P*. *larvae*, consequently increasing the number of options for the control of this pathogen.

Since the discovery of the first lysin for *P*. *larvae* species in 2015^17^ many other lysins were annotated^23,26–29^, However, no CBD has been identified so far. In order to understand the similarity of PlyPl23 with other *P*. *larvae* phage lysins a BLASTp analysis was performed, revealing high homologies at the protein level with 17 other *P*. *larvae* phage lysins. Curiously, no homologies were found with lysins affecting other *Bacillus* species. This means that the *P*. *larvae* cell wall might be different from the cell walls of other species or/and that CBD targeting *P*. *larvae* binds to uncommon receptors on the cell wall.

Multiple sequence alignment of all the fetched *Paenibacillus* phage lysins using Praline^18^ (Fig. 3 and S1) revealed a well conserved group composed of lysins from 9 phages (phiIBB_Pl23, Harrison, Xenia, Diva, Lily, Fern, Diane, HB10c2 and Redbud) all of them with an Amidase_2 at the N terminus. PG1 lysin is also an Amidase_2 lysin, however less related to the other nine, making the bridge between the Amidase_2 group and the Glucosaminidase, Amidase_3 and Glyco_hydro_25 lysins. The lysin of phiPB (a phage infecting *P*. *polymyxa*) has a C-terminal sequence quite different from the PlyPl23, showing that they target different receptors. This might be correlated with different cell wall composition/structures. These differences can explain the reduced/lack of activity (binding) in *P*. *larvae* by phiBP^25^ lysin (CBD) and similarly, the lack of activity to *P*. *polymyxa* by PlyPl23.

A closer look on the alignment of the conserved lysins group (Fig. 3b) reveals a high similarity between them (sequence identity = 0.95), with only a few single amino acid mutations. Even so, these few amino acids are enough to cause modifications on the secondary structure of the CBD (as predicted by Praline^18^, Fig. 4) which leads to differences on the lysin activity, either on efficiency or/and on specificity. Six of the 9 lysins start with a double methionine amino acid and only PlyPl23 and the lysin from the Harrison phage have a cysteine on the second last position (which seems to be an insertion when compared to the other phage lysins, Fig. 4), a residue that we showed to be decisive in binding to ERIC *IV* strains. This definitely contributed to the very low activity of Xenia’s phage lysin PlyPalA^23^ towards this genotype.

With the exception of the first methionine, the lysin pairs from phages phiIBB_Pl23/Harrison, Xenia/Diva and HB10c2/Redbud have exactly the same peptide sequence (Figs 3b and S1) and consequently the same CBD sequence (Fig. 4). Lysins from Jimmer1 and Abouo are also well conserved with only some point mutations. These pairs are very well grouped on the tree diagram (Fig. 3a). Such conserved sequences, even at the usual non-conserved C-terminus, show that many CBDs targeting *P*. *larvae* are quite conserved. This reinforces the importance of this study. The findings described herein will enable the identification of further domains, improving this way the development of new tools to detect and control *P*. *larvae* based on CBDs.

In conclusion, we have cloned the C-terminus of PlyPl23 lysin fused to a GFP and found that it is able to decorate *P*. *larvae* cells proving the existence of a functional CBD, the first identified so far targeting this pathogen. The CBD is able to bind *P*. *larvae* strains from all ERIC genotypes, presenting a broader spectrum than its corresponding phage, but still with high specificity at the species level. Such specificity, together with the intrinsic binding affinity of the CBDs, shows a high potential as a bio-recognition element in the development of a diagnostic tool and also as a GPS for unspecific protein antimicrobials. Importantly, the non-interference of HL in CBD binding supports its field application.

Sequential deletions of the CBCF enabled the identification of the CBD with a length of 63 amino acids, composed of residues E161 to C223 that showed to be essential for ERIC *IV* genotype binding.

Alignment of the existing *Paenibacillus* lysins’ sequences revealed that the CBDs are well conserved among Amidase_2 lysins and consequently this study will enable an easy identification of such domains from now on, increasing the pool of available CBDs targeting *P*. *larvae* in a near future.

## Materials and Methods

### Bioinformatic analysis

PlyPl23 (NCBI Reference Sequence: YP_008320357) was previously identified as a lysin belonging to the Amidase_2 family, with a N-acetylmuramoyl-L-alanine amidase catalytic domain at its N-terminus, encoded by the *Siphoviridae P. larvae* phage phiIBB_Pl23 (NCBI Reference Sequence: NC_021865)^17^. The search for additional functional domains was carried using Motif Search (http://www.genome.jp/tools/motif), InterProScan^30^ and HHpred^31^. A BLASTp^32^ analysis was carried to find homologs on the non-redundant protein database and Praline^18^ was used for multiple sequence alignment, predicted secondary structure and tree diagram with an E-value cut-off of 1E-5. Phyre2^33^ was used to predict the protein structure using the “Intense” modelling mode and the image obtained with iCn3D Structure Viewer^34^ available at the NCBI website (https://www.ncbi.nlm.nih.gov/Structure/icn3d/full.html).

### Cloning

The *Aequorea coerulescens* GFP gene was inserted into the plasmid pET28a(+) (Novagen), between the *NdeI* and *BamHI* restriction sites conserving the plasmid N-terminal hexa-histidine (His)-tag sequence and originating the pET_GFP plasmid. Primers were designed (Table 1) to obtain different fragments of the PlyPl23 lysin C-terminus (further referred as the cell binding containing fragment - CBCF). These primers enabled to truncate the CBCF, first on the N-terminus (conserving the C-terminus) and then at the C-terminus (conserving the N-terminus). Primer melting temperatures were calculated using OligoCalc^35^. The fragments were amplified with Phusion DNA Polymerase (ThermoFisher Scientific) with the PlyPl23 plasmid^17^ as DNA template and digested with the restriction enzymes *SacI* and *XhoI*. The digested fragments were inserted into the pET_GFP (in order to fuse them with the GFP upstream, at the N-terminus) and ligated with the T4 ligase (ThermoFisher Scientific) to obtain the different constructions (pET_GFP-CBCFx), further used to transform *E. coli* TOP10 competent cells (Invitrogen). Colonies were screened through colony PCR and positives used for plasmid extraction and further confirmation through Sanger sequencing. Correct pET_GFP-CBCFx plasmids were used to transform competent *E*. *coli* BL21 (DE3).

### Expression, purification and quantification

Expression of the different peptides was performed as described before^17^. Briefly, cells harboring each recombinant plasmid were grown at 37 °C in Lysogeny Broth (LB) medium supplemented with 50 μg.ml-1 of kanamycin until reaching an optical density at 620 nm (OD_620nm_) of 0.600. Recombinant protein expression induced with isopropyl-β-D-thiogalactopyranoside (IPTG) at 1 mM final concentration was carried overnight at 16 °C, 150 rpm. Cells were harvested by centrifugation (9000 g, 15 min) and further resuspended in lysis buffer (20 mM NaH_2_PO_4_, 500 mM sodium chloride, 10 mM imidazole, pH 7.4). Cell disruption was made by thaw-freezing (3 cycles, from −80 °C to room temperature) followed by a 5 min sonication (Cole-Parmer Ultrasonic Processor) for 10 cycles (30 s ON, 30 s OFF), 40% amplitude. Soluble cell-free extracts were separated by centrifugation, filtered, and loaded on a 1 mL HisPur™ Ni-NTA Resin (Thermo Scientific) stacked in a Polypropylene column (Qiagen). After two washing steps with protein-dependent imidazole concentrations (lysis buffer supplemented with 20 mM imidazole in the first wash and 40 mM imidazole in the second wash) the protein was eluted with 300 mM imidazole. Samples of each fraction were analyzed by SDS-PAGE.

The purified proteins were concentrated and dialyzed against the storage buffer (10 mM Tris-HCl, pH = 7.0) using the centrifugal filters Amicon Ultra—0.5 mL (Millipore) and stored at 4 °C. Protein concentration was determined using the BCA Protein Assay Kit (Thermo Scientific) with bovine serum albumin (BSA) as standard.

### Bacterial strains

The phage host, *P*. *larvae* Pl02-23, belonging to the enterobacterial repetitive intergenic consensus (ERIC) genotype *I*, was used in the binding tests as well other reference type *P*. *larvae* strains: LMG 9820, CCUG 48972 and CUCG 48973, LMG 15974 and LMG 16252, LMG 16247 and LMG 16250 reference strains representing genotypes ERIC *I*, *II*, *III* and *IV*, respectively. Typing of *P*. *larvae* strains was performed as reported by Genersch and Otten^36^. Other non-*P*. *larvae* strains were also used: *Paenibacillus polymyxa* (LMG 13294), *Paenibacillus alvei* (LMG 13253), *Lactobacillus pentosus* (CECT 4023), *Lactobacillus rhamnosus* (CECT 288), *Lactobacillus paracasei* (CECT 277), *Lactobacillus casei* (CECT 5275), *Lactobacillus acidophilus* (CECT 4356), *Bacillus subtilis* (DSMZ 10), *Bacillus coagulans* (CECT 12), *Bacillus cereus* (CEB collection). The first-instar larvae commensal bacterial strains *Lactobacillus kunkeei* (LMG 18925) and *Parasaccharibacter apium* alpha 2.2 (strain C6)^37^ were also tested.

The CCUG strains were obtained from the Culture Collection of the Goteborg University, LMG from the BCCM - Belgian Coordinated Collections of Microorganisms, DSMZ from the Deutsche Sammlung von Mikroorganismen und Zellkulturen GmbH and CECT from the Colección Española de Cultivos Tipo.

### Cell wall binding assay and fluorescence microscopy

The binding ability of the different truncations of the CBCF fused to GFP was inferred by fluorescence microscopy observations of *P*. *larvae* cells after incubation with the fused proteins. GFP alone was used as a negative control. To prepare the samples for observation, bacteria were recovered from MYPGP agar plates incubated overnight (37 °C, 5% CO_2_) and harvested by centrifugation (9000 × g, 5 min) to obtain a concentration of 10^9^ CFU.mL^−1^. The pellet was re-suspended in the same volume of NaCl and then mixed 1:1 (v/v) with 20 μM of GFP-CBCFx diluted in Tris-HCl 10 mM pH = 7 and incubated for 10 minutes at room temperature. Cells were washed twice (9000 ×g, 5 min) in 1 mL 0.9% NaCl, and the pellet was re-suspended in 50 μL 0.9% NaCl. A volume of 10 μL of the resultant suspension was observed at the fluorescence microscope (Olympus BX51, Magnitude 1000×) in bright field and under the FITC filter.

Bee larvae artificially contaminated with *P*. *larvae* was used to assess the influence of the larvae matrix on the binding ability of the GFP-CBCFx. For that, n = 14 (1^st^, 2^nd^ and 3^rd^ instar) larvae were grafted from combs and washed twice in distilled water. Larvae were pelleted by centrifugation (9000 ×g, 5 min), suspended in 25 μl 0.9% NaCl and homogenized with a pestle. A volume of 10 μL (10^7^ CFU.mL^−1^) of Pl02-23; *Lactobacillus* *kunkeei*; *Parasaccharibacter* *apium* suspensions was added to each replicate of homogenized larvae (HL). Afterwards, 10 μl of these homogenized larvae (HL) samples were mixed 1:1 (v/v) with 20 μM of GFP-CBCFx and submitted to the cell wall binding assay.

### Accession number

The NCBI Reference Sequence of the lysin PlyPl23 is YP_008320357.

### Ethical approval

This article does not contain any studies with human participants or animals performed by any of the authors.

## Supplementary information

Figure S1
