# Supplementary material for: Identification of the first endolysin Cell Binding Domain (CBD) targeting Paenibacillus larvae
Source: Sci Rep. 2019 Feb 22;9:2568. doi: 10.1038/s41598-019-39097-2 (PMC6385185; doi:10.1038/s41598-019-39097-2)
Supplement: Supplementary file 1 — Figure S1 [file 41598_2019_39097_MOESM1_ESM.pdf]

Identification of the first endolysin Cell Binding Domain (CBD) targeting *Paenibacillus larvae*

Sílvio B Santos[#] and Ana Oliveira*[#], Luís D. R. Melo and Joana Azeredo

CEB - Centre of Biological Engineering, LIBRO - Laboratório de Investigação em Biofilmes Rosário Oliveira, University of Minho, 4710-057 Braga, Portugal.

*Corresponding Author: anaoliveira@deb.uminho.pt, Tel. (+351) 253 601968, Fax (+351) 253 604 429.

[#]Both authors contributed equally to this work

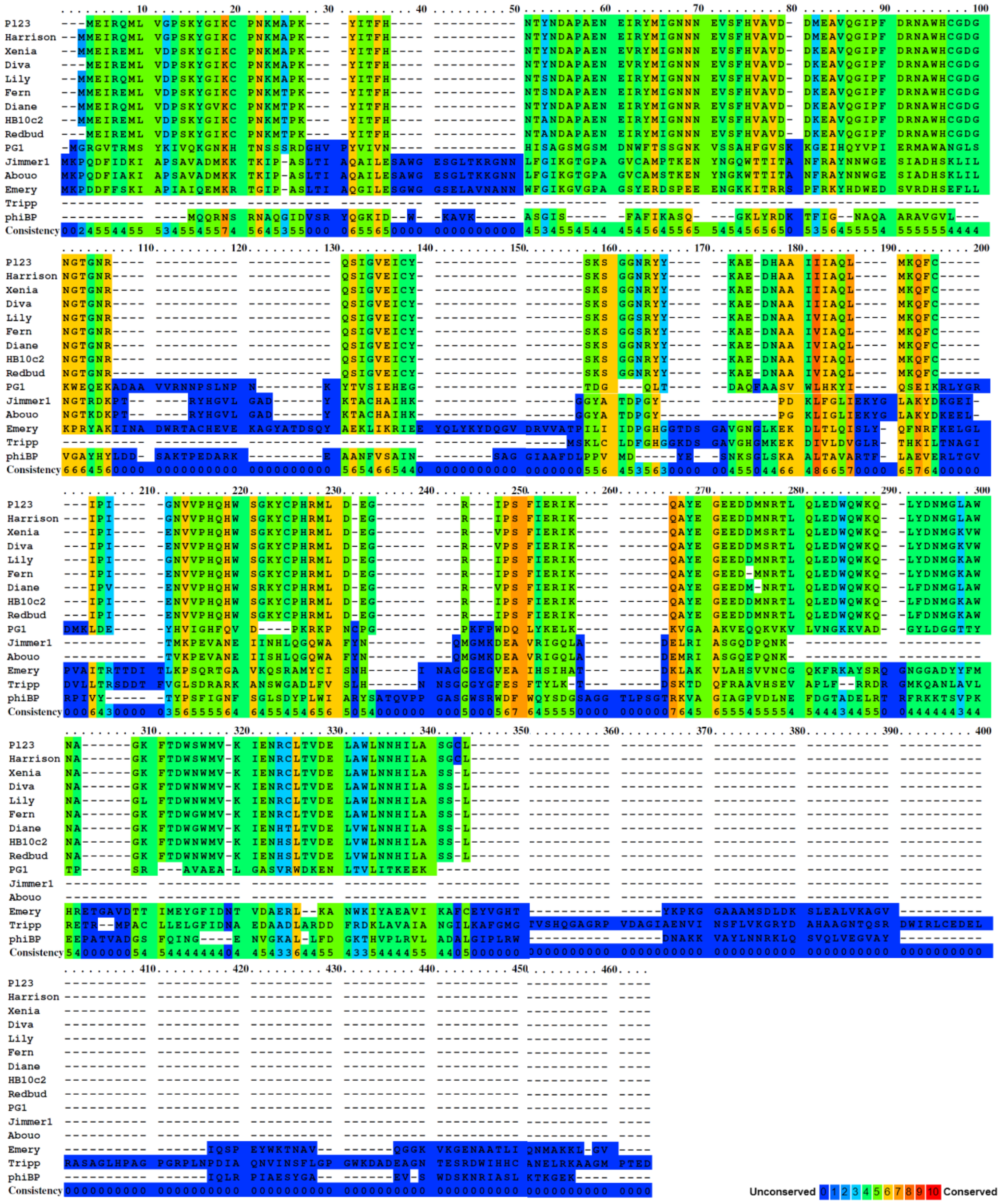

Fig. S1 Colour-coded scheme for amino acid conservation of the multiple sequence alignment of all the *Paenibacillus* phage lysins (15 lysins from 25 proteomes) using Praline [18]. The scoring scheme works from 0 for the least conserved alignment position, up to 10 for the most conserved alignment position.
